# Biosignatures for Parkinson’s Disease and Atypical Parkinsonian Disorders Patients

**DOI:** 10.1371/journal.pone.0043595

**Published:** 2012-08-27

**Authors:** Judith A. Potashkin, Jose A. Santiago, Bernard M. Ravina, Arthur Watts, Alexey A. Leontovich

**Affiliations:** 1 The Cellular and Molecular Pharmacology Department, Rosalind Franklin University of Medicine and Science, The Chicago Medical School, North Chicago, Illinois, United States of America; 2 Neurology Department, University of Rochester School of Medicine, Rochester, New York, United States of America; 3 Biogen Idec, Cambridge, Massachusetts, United States of America; 4 Department of Biostatistics, University of Rochester School of Medicine, Rochester, New York, United States of America; 5 Division of Biomedical Statistics and Informatics, Mayo Clinic, Rochester, Minnesota, United States of America; University of Nebraska Medical Center, United States of America

## Abstract

Diagnosis of Parkinson’ disease (PD) carries a high misdiagnosis rate due to failure to recognize atypical parkinsonian disorders (APD). Usually by the time of diagnosis greater than 60% of the neurons in the substantia nigra are dead. Therefore, early detection would be beneficial so that therapeutic intervention may be initiated early in the disease process. We used splice variant-specific microarrays to identify mRNAs whose expression is altered in peripheral blood of early-stage PD patients compared to healthy and neurodegenerative disease controls. Quantitative polymerase chain reaction assays were used to validate splice variant transcripts in independent sample sets. Here we report a PD signature used to classify blinded samples with 90% sensitivity and 94% specificity and an APD signature that resulted in a diagnosis with 95% sensitivity and 94% specificity. This study provides the first discriminant functions with coherent diagnostic signatures for PD and APD. Analysis of the PD biomarkers identified a regulatory network with nodes centered on the transcription factors HNF4A and TNF, which have been implicated in insulin regulation.

## Introduction

Parkinson’s disease (PD) is the second most common neurodegenerative disease. Approximately 95% of cases of PD are idiopathic most likely caused by environmental factors and genetic susceptibility. Unfortunately by the time of diagnosis most of the dopaminergic neurons in the substantia nigra are dead. Diagnosis of PD is based on classical motor symptoms including resting tremor, rigidity, bradykinesia and postural instability. Despite clinical criteria for PD there remains a high rate of misdiagnosis with atypical parkinsonian disorders (APD), such as progressive supranuclear palsy (PSP) and multiple system atrophy (MSA) [1]. APD accounts for 10–20% of individuals with parkinsonism [2]. MSA is a sporadic neurodegenerative disease characterized by parkinsonian symptoms, cerebellar ataxia and autonomic dysfunction. Like PD, MSA is an alpha-synucleinopathy in which glial cytoplasmic inclusions most likely play a role in the pathogenesis of the disease. PSP is characterized by supranuclear palsy, postural instability, ophthalmoplegia and mild dementia. Accumulation of neurofibrillary tangles composed of tau protein is a common pathological feature of PSP. Despite the distinct pathological features there is phenotypic overlap between these disorders. Since early diagnosis is difficult, minimally invasive biomarkers capable of distinguishing PD from APD would facilitate clinical care and clinical research.

Messenger RNA (mRNA) transcripts are excellent candidates for diagnostic biomarkers since very small quantities may be amplified and quantified by quantitative polymerase chain reaction (qPCR). Numerous studies have examined changes in global gene expression in postmortem brains of PD patients and these have been analyzed in a meta-genome-wide expression study (GWES) [3]. In these studies PD patients exhibited changes in expression of genes related to dopaminergic neurotransmission, synaptic function, electron transport, ubiquitin-proteasomal system, cytoskeletal maintenance, cell cycle and adhesion. Standard microarrays were used in these studies and, therefore, changes in transcription and RNA stability were assessed. In addition to these mechanisms of regulation of gene expression, alternative splicing responds rapidly to environmental factors to produce several mRNAs from a single pre-mRNA. It has been estimated that 92–94% of human pre-mRNAs are alternatively spliced [4]. Many neurological diseases are associated with abnormalities in the regulation of splicing including autosomal recessive juvenile parkinsonism (AR-JP) [5] and frontotemporal dementia and Parkinsonism linked to chromosome 17 (FTDP-17) [6], [7]. In addition, DJ-1, which plays a role in the development of some forms of familial PD, has been implicated in tyrosine hydroxylase splicing by inhibiting the sumoylation of the splicing factor PTB [8]. The deregulation of synphilin-1 splicing also occurs in the brains of PD patients [9]. Mitochondrial damage, which plays a role in PD, disrupts splicing regulation in neurons [10]. A few studies also examined changes in splicing in animal models of PD. In an MPTP mouse model, overexpression of one splice variant, ache-r, in the brain was protective, whereas overexpression of another variant, ache-s, enhanced the development of Parkinsonism [11]. In another study, the expression of splice variants of fosB and rgs9 was disrupted in the striatum and/or substantia nigra pars compacta of MPTP-treated Parkinsonian mice compared to controls [12].

It is clear from the above studies that the loss of nigrostriatal dopamine neurons in PD correlates with changes in splicing within the brain. Brain tissue, however, is not a useful source for PD biomarkers and RNA is often degraded in post-mortem tissue. In contrast, blood biomarkers are useful because they are non-invasive. In this regard, it is clear that the immune system responds to changes in dopamine [13]. This is not unexpected since catecholamines are synthesized from tyrosine in lymphocytes and macrophages [14]. Dopamine receptors are expressed in T lymphocytes, monocytes, neutrophils, eosinophils, B cells and natural killer cells [15]. Dopamine affects the activity of regulatory T cells [16]. A “brain-to-T cell” pathway has been proposed to explain how peripheral T lymphocytes might respond to dopamine in the brain based on the fact that T lymphoblasts can cross the blood-brain barrier [17]. Dopamine transporters are also expressed in lymphocytes [18]. Dopamine biosynthesis and signaling is disrupted in the blood of PD patients [19], [20], [21], [22]. In addition, PD patients have altered mitochondrial function in the blood similar to that seen in the brain [23], [24], [25].

In an earlier study, total mRNA abundance in whole blood was assayed using standard microarrays in order to identify transcripts associated with risk of PD [26]. In later studies, the expression of several additional genes was shown to be dysregulated in blood cells of patients compared to controls [27], [28]. In these studies, splice variant-specific biomarkers may have been missed since total mRNA was measured. Since environmental factors play an important role in idiopathic PD, it is highly likely that splice variants in blood may provide a rich source of biomarkers potentially more sensitive than gene expression profiling. In this regard, splice variants of parkin and other transcripts were found to be dysregulated in leukocytes of PD patients compared to healthy controls (HC) [29], [30]. In addition, the splicing factor SRRM2 is dysregulated in whole blood of PD patients [31].

Here, we identify and validate biosignatures for PD and APD composed of splice variant biomarkers in blood whose expression is altered in patients compared to controls. Network analysis of the PD biomarkers reveals a network centered on the transcription factors HNF4A and TNF, which have been implicated in insulin regulation.

## Results

### Biomarker Discovery and Validation

Samples used in this study came from early stage PD patients (Hoehn & Yahr scale stage 1 and 2) and age-matched healthy HC, MSA and PSP controls who were enrolled in the Prognostic Biomarker Study (#NCT00653783) [32]. Information about the study participants is provided in Table 1 and inclusion/exclusion criteria used for participants of the study and the diagnosis of PD, MSA and PSA are provided in Table S1. Clinical diagnosis of PD was based on the United Kingdom Parkinson’s Disease Society Brain Bank criteria [33]. A diagnosis of probable MSA was based on Consensus Criteria [34] and probable PSP based on NINDS-PSP Criteria [35].

**Table 1:** Information about study participants.

	PD			HC			MSA			PSP			
Patient cohort	T1	T2	Test	T1	T2	Test	T1	T2	Test	T1	T2	Test	Total
Number	19	12	20	11	10	18	4	6	7	5	5	7	124
Median age	61	60	58	65	55	57	64	63	63	74	77	67	NA
Average age	63	64	61	65	56	61	65	62	64	72	72	68	NA
% Female	37	8	10	55	40	77	50	63	37	60	20	43	NA
% Male	63	92	90	45	60	33	50	67	63	40	80	57	NA
L-dopa	19	12	19	0	0	0	4	6	7	3	5	6	81
Diabetes	0	0	0	2	0	2	0	1	1	0	1	0	7
Hoehn & Yahr stage	2	2	2	NA	NA	NA	NA	NA	NA	NA	NA	NA	NA

HC is healthy control, PD is Parkinson’s disease, MSA is multiple system atrophy, PSP is progressive supranuclear palsy patients, T1 is training set 1, T2 is training set 2 and NA is not applicable.

**Figure 1:**
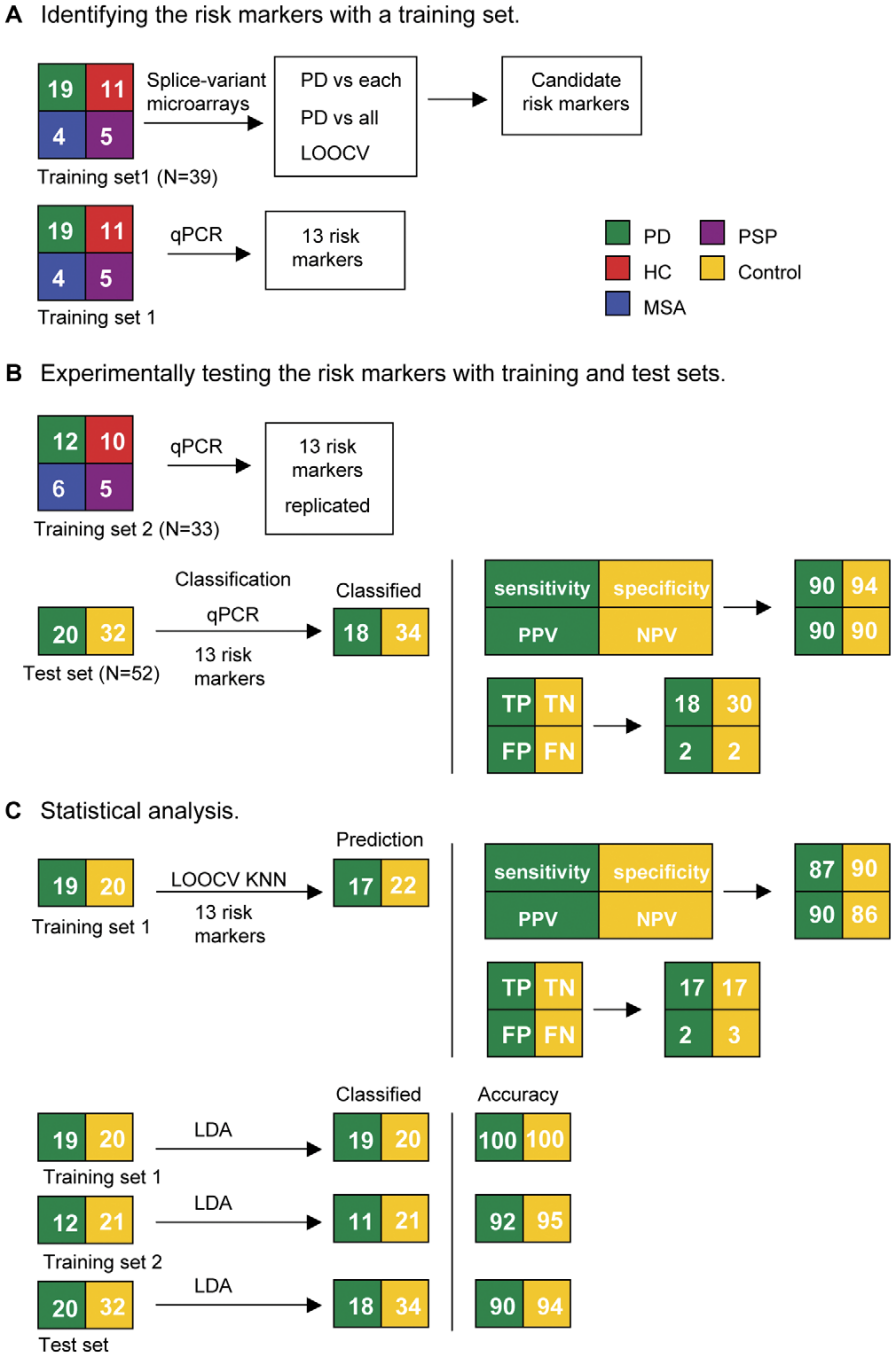
Design of workflow used to identify splice variant-specific risk markers of PD. The numbers inside the boxes correspond to the number (N) of samples. Samples from PD patients were randomly chosen to be part of the training or test sets of samples. (A) Identifying the PD risk markers using a training set. In order to identify putative biomarkers, the training set (training set 1) was used in the microarray screen. The data from the microarray analysis was analyzed by three methods in order to identify markers with good specificity, sensitivity and predictive accuracy. PD patients were compared to each control group separately, compared to pooled controls as a single group and using LOOCV. Splice variants that were up- or down-regulated by 2-fold (P<0.05) in PD patients were considered candidate risk markers. The candidate risk markers were manually curated to include those that may play a role in PD based on pathway and disease analysis (Ingenuity Systems software) and to exclude those for which primers could not be designed or could not be detected by qPCR. Thirteen of the risk markers were replicated by qPCR (Training set 1). (B) Experimentally testing the PD risk markers with training and test sets. The 13 risk markers were validated in two independent test sets (training set 2 and test set) using qPCR. Clinical diagnosis of the participants was based on neurological exam. (C) Statistical analysis. LOOCV KNN was used to determine the predictive accuracy of the samples from the training set (training set 1). In addition, linear discriminant analysis was used to test the predictive accuracy on the training and test sets. PD is Parkinson’s disease patients represented in green, HC is healthy controls represented in red, MSA is multiple system atrophy controls represented in blue, PSP is progressive supranuclear palsy controls represented in purple and Control is HC+MSA+PSP controls represented in yellow. TP = true positive; TN = true negative; FP = false positive; FN = false negative; PPV = positive predictive value; NPV = negative predictive value.

**Figure 2:**
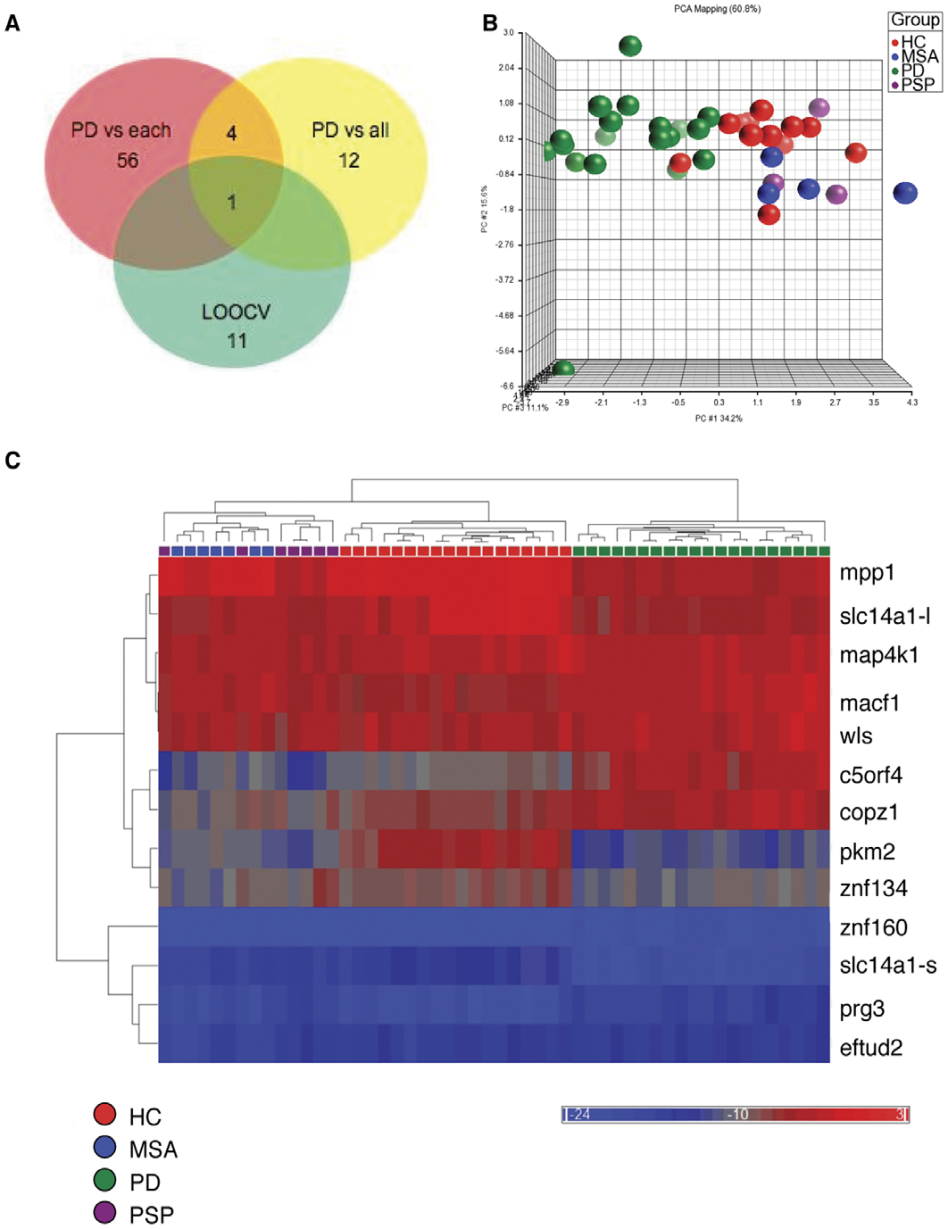
Heat map and PCA plot of the PD risk markers. (A) Venn diagram of splice events that were differentially expressed in PD patients compared to controls. PD patients were compared to each control group (HC, MSA or PSP) individually or a pooled control group that included HC, MSA and PSP participants. (B) PCA analysis of the data from the microarrays of the risk markers. HC is healthy control represented in red, PD is Parkinson’s disease patient represented in green, MSA is multiple system atrophy control represented in blue and PSP is progressive supranuclear palsy control represented in purple. (C) Heat map of the data from the qPCR assay of the risk markers analyzed using the ΔΔCt method. Each column in the heat map corresponds to a PD patient or a control. Each row represents the relative level of abundance of a single splice variant. Each splice variant is denoted by the name of the mRNA. Color scales representing splice variant expression with red representing high abundance relative to the mean abundance; blue representing low abundance relative to the mean abundance; and gray representing no significant change in abundance level between the sample and control. Gene expression microarray data has been deposited at Gene Expression Omnibus under accession number GSE34287.

**Figure 3:**
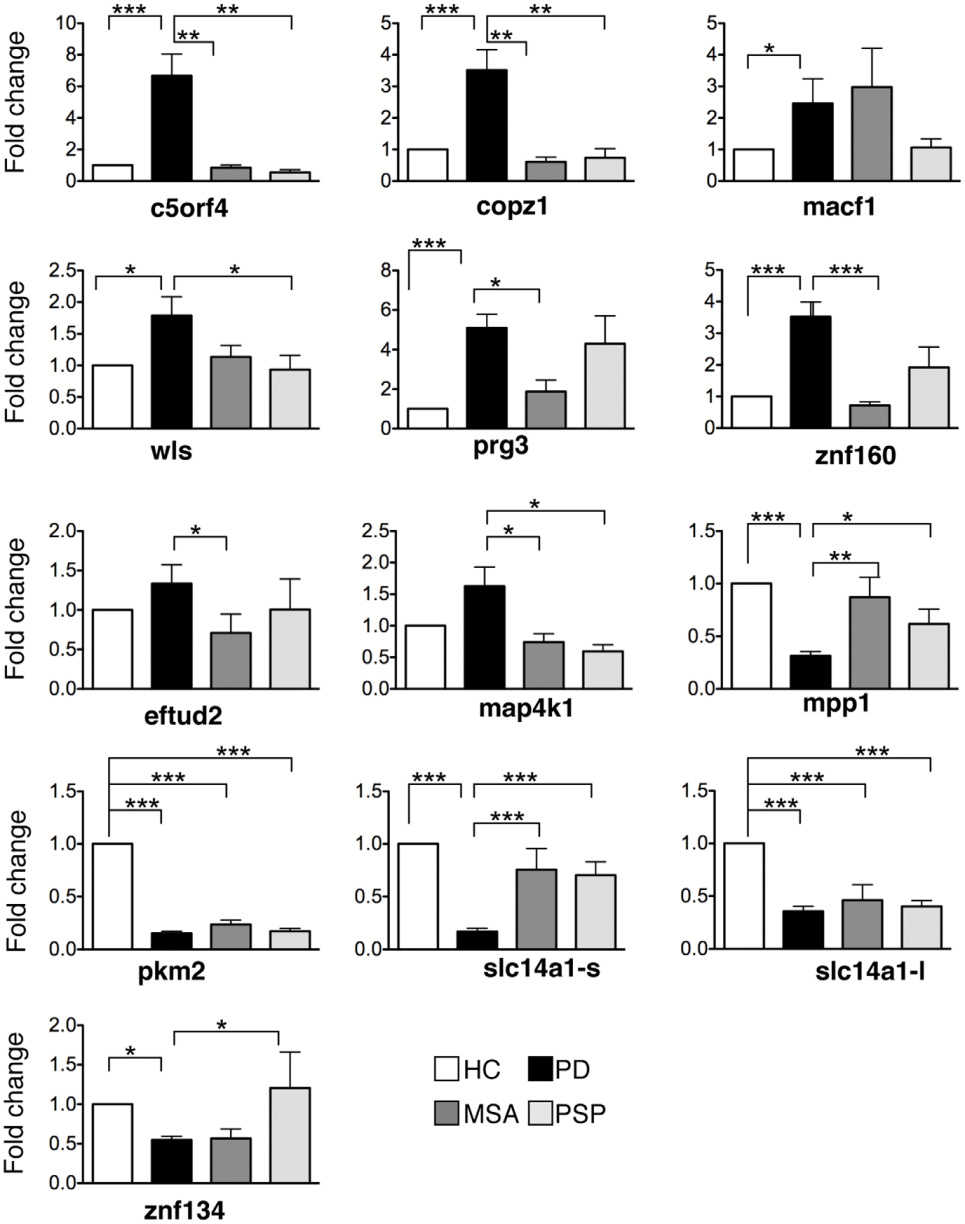
Relative mRNA quantification graphs of the risk markers comparing PD patients with each control group. A one-way ANOVA and tukey-kramer post-hoc analysis was used to compare PD patients with HC, MSA and PSP controls separately. Fold change values relative to a calibrator are displayed with error bars indicating SEM. Gapdh mRNA was used as a reference gene and HC as a calibrator. PD is Parkinson’s disease, HC is healthy control, MSA is multiple system atrophy control and PSP is progressive supranuclear palsy control. *p<0.01, **p<0.005, ***p<0.001 and ****p<0.0001.

In order to identify a splice variant-specific profile associated with PD we probed cDNA prepared from RNA extracted from whole blood of participants with 257,319 probes on microarrays designed to monitor splicing events (Fig. 1A, training set 1). Transcripts (10,563) that were differentially expressed between any two groups at least 2-fold were analyzed further as putative biomarkers. The data from the microarrays was analyzed to identify disease markers with good predictive accuracy. Splice variants expressed in PD patients were compared to each control group individually to identify 61 putative markers and compared to a pool containing all of the control groups to identify 12 additional candidates (Fig. 2A). In a third analysis, leave-one-out-cross-validation (LOOCV) was used to optimize the accuracy of prognostic prediction of the splice variants, which produced 11 additional candidates. Candidate risk markers were prioritized for validation based on the role they may play in PD etiology or progression. Some candidates were eliminated due to technical difficulty in the design of splice variant-specific primers or their abundance was determined to be insufficient to be reliably detected by qPCR. Thirteen splice variants met these criteria and were validated in qPCR assays (training sets 1 and 2, Figs. 1A, 1B, 3 and S2A). The final PD classifier included c5orf4, wls, macf1, prg3, eftud2, pkm2, slc14a1-s, slc14a1-l, mpp1, copz1, znf160, map4k1 and znf134.

Principal components analysis (PCA) on the microarray data and cluster analysis of the PCR data showed that PD patients can be separated from controls using the 13 biomarkers (Fig. 2B and 2C). In order to identify splice events whose abundance correlates with the binary diagnostic categories (PD vs. controls), we calculated the Pearson correlation coefficients (r). The frequency distribution of 257,319 r values (representing splice events) was plotted on a histogram to identify candidate risk markers (Fig. S3). Twelve of the markers have r values above the 95th percentile or below the 10th percentile indicating a non-random association.

Samples from HC were compared to those from PD, MSA and PSP patients. The results showed that macf1, mpp1, pkm2, and slc14a1-l are expressed differentially in healthy individuals compared to diseased participants (Fig. S4). In order to determine whether expression of any of the risk factors correlated with dopamine therapy, Pearson correlation coefficients were determined. The expression of none of the markers correlated with dopamine therapy except slc14a1-s (r = 0.66, p = 0.003).

### Testing the Prediction Accuracy of the PD Biomarkers

To assess the prediction accuracy of the PD markers in the classification of the training set, we carried out a LOOCV (Fig. 1C). The prediction accuracy was 88%, with 87% sensitivity (17 out of 20) and 90% specificity (17 out of 19). Experimental results from the test set indicated that PD patients were identified with 90% sensitivity (p = 0.0001) and 94% specificity (p = 0.00001) in accordance with the clinical diagnosis (Fig. 1B). These results indicate that with the 13 markers, individuals could be identified as PD or non-PD.

**Figure 4:**
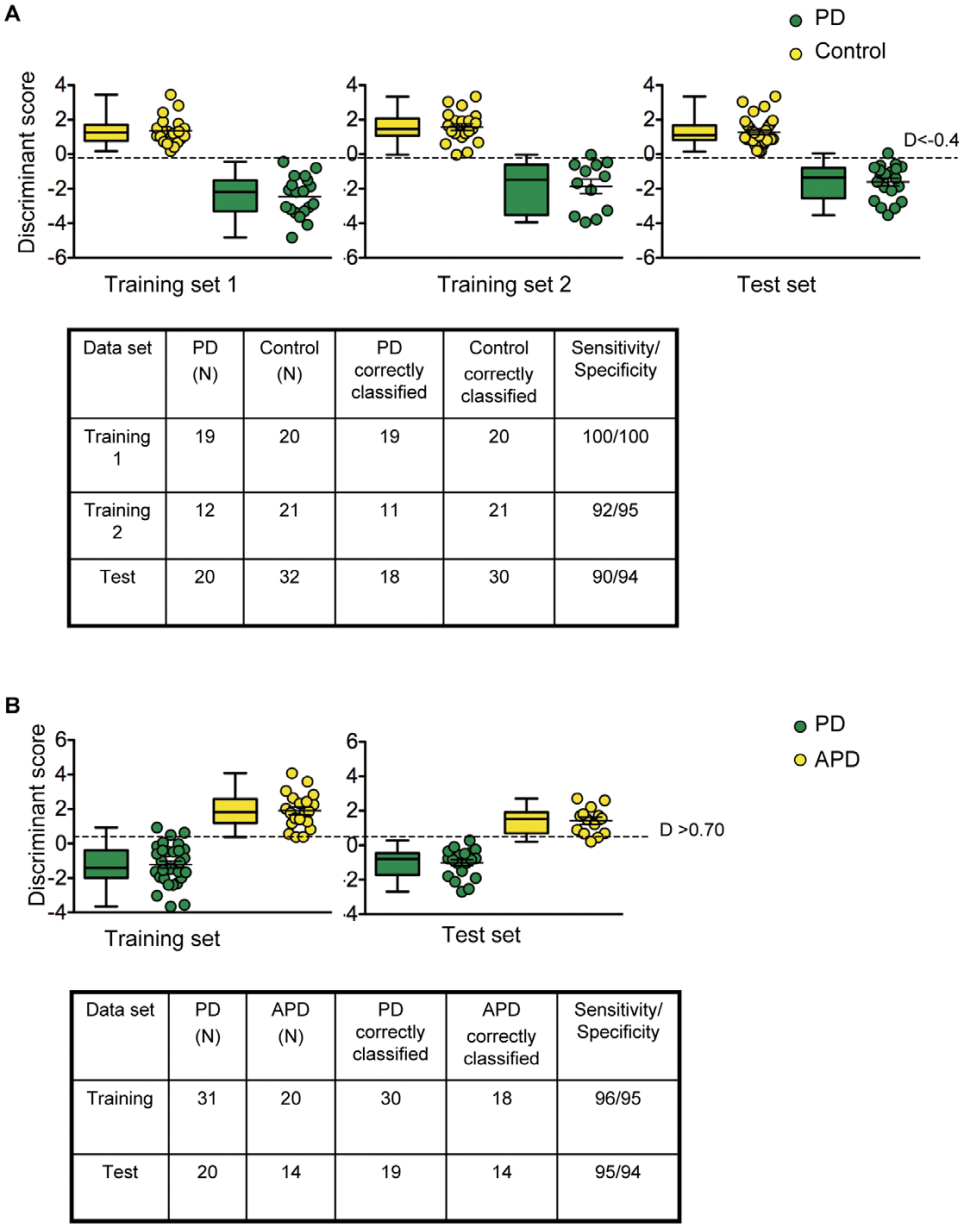
Linear discriminant analysis of the biomarkers. (A) Discriminant scores of PD samples (green) are compared to controls (HC, PSP and MSA, yellow) using the 13 PD biomarkers. (B) Discriminant scores of PD samples (green) are compared to APD (PSP and MSA, yellow) using the 8 APD biomarkers.

In order to build a prediction model with the highest possible accuracy, we performed a linear discriminant analysis to determine which of the biomarkers best discriminate between PD and controls using the expression values for each biomarker. Discriminant analysis on the training and test sets showed that the set of 13 markers resulted in an overall sensitivity of 94% and a specificity of 96%. (Figs. 1C and 4A). We calculated the discriminant function coefficients for each marker (Table S3). The discriminant function revealed a significant association between groups and all predictors, accounting for 85% of between group variability (Table S4). The four most significant predictors were c5orf4 (0.69), mpp1 (0.50), macf1 (0.42), and copz1 (0.40) (standardized coefficients). Based on this analysis, the canonical PD discriminant equation is
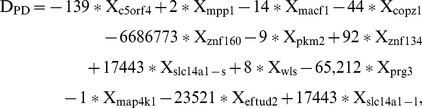
where D_PD_ is the discriminant score value (raw canonical coefficients, Table S3) and X_i_ is the mRNA expression level of each biomarker. We classified cases that give a D_PD_ value below the cutting point (D≤−0.4) as PD and those above as non-PD (Fig. 4A).

Linear discriminant analysis was also used to determine the predictive accuracy of the biosignature to discriminate between PD and APD patients and HC for all 124 participants. Based on this analysis, PD patients were identified with 94% sensitivity and 96% specificity, APD patients with 91% sensitivity and 97% specificity and HC controls with 69% sensitivity and 87% specificity (Table S5).

### Identifying and Testing the Prediction Accuracy of APD Biomarkers

Principle components analysis and heat map analysis shows that samples from PD patients cluster separately from APD patients using the 13 markers (Fig. S5). To identify the markers with the highest possible prediction accuracy for distinguishing PD from APD, we implemented a forward stepwise linear discriminant analysis (LDA) to build a prediction model. LDA was initially performed with samples from 31 PD and 20 APD patients (training set) using 13 PD biomarkers. The Wilk’s lambda criterion was used to determine if a biomarker became part of the final prediction model. Implementation of the LDA on the training set revealed that the 8 markers (copz1, c5orf4, mpp1, macf1, wls, slc14a1-l, znf134 and map4k1) accurately distinguish PD from APD with 96% sensitivity and 95% specificity (Figure 4B). Relative mRNA expression for the biomarkers shows that c5orf4, copz1, macf1, and wls are up-regulated in PD whereas mpp1 is down-regulated (Fig. S2B). The strongest predictors were c5orf4 (0.99), macf1 (0.78), mpp1 (0.57) and copz1 (0.54), according to the standardized coefficients for the canonical variables (Table S6). Based on this analysis, the resulting APD discriminant equation is
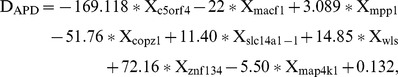
where D_APD_ is the discriminating value (raw canonical coefficients, Table S6) and X_i_ is the mRNA expression level of each biomarker. We classified cases that give a D_APD_ value above the cutting point (D≥0.7) as APD and those below as PD (Fig. 4B).

To determine whether the molecular signature of 8 risk markers could accurately discriminate PD from APD, we applied the discriminant function to a test set consisting of 20 PD and 14 APD patients. Mahalanobis distance between each case and the centroid of the group was evaluated and no significant deviation was observed. Multicollinearity is not a problem in the final prediction model since the tolerance values for all predictors is higher than 0.10 (Table S7). To assess the robustness of our prediction model, the canonical correlation was evaluated. The eigenvalue is 1.99 and the canonical correlation accounts for 82% of the variance. The Wilk’s lambda value was statistically significant (0.33, P<10^−04^, Table S8). Using the discriminant analysis consisting of 8 APD markers, 19 samples out of 20 were classified as PD whereas the remaining 14 were classified as APD with 95% sensitivity and 94% specificity in accordance with the clinical diagnosis (Figure 4B).

To determine the predictive accuracy of the APD biosignature to discriminate PD from APD we implemented a linear discriminant analysis using the gene expression data from all 85 patients. Based on this analysis, PD patients can be distinguished from all controls with 96% sensitivity and 90% specificity and from APD patients with 94% sensitivity and 96% specificity (Table S9).

### Assessing the Biological Relevance of the Biomarkers

**Figure 5:**
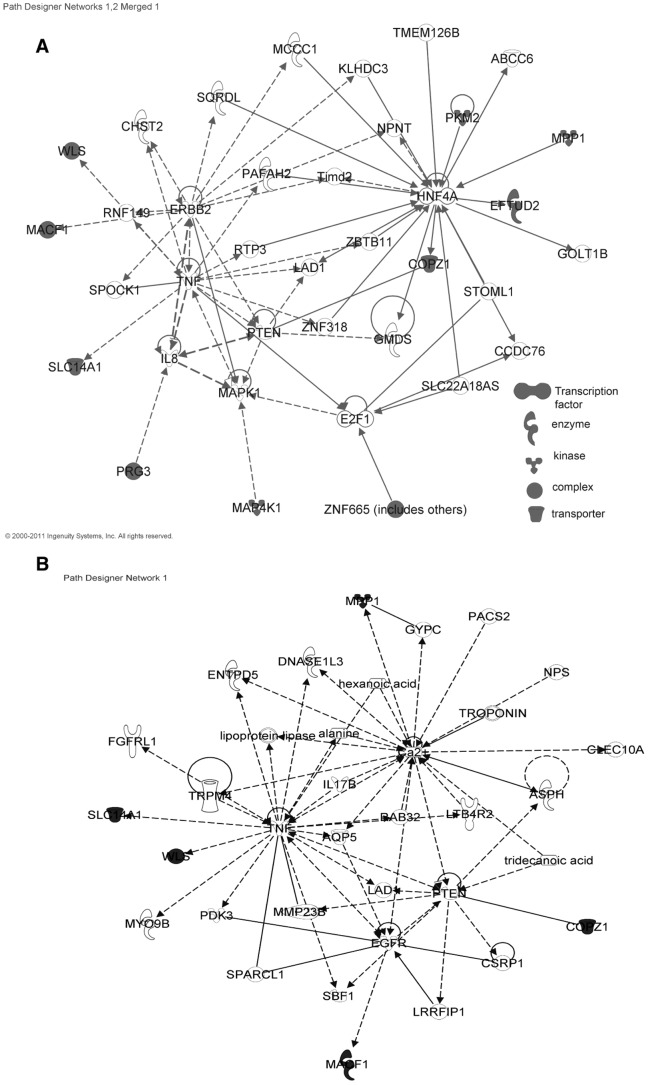
Regulatory gene and protein interaction networks. (A) Network of the PD biomarkers. (B) Network of the APD biomarkers. Computational molecular interaction network prediction based on genes and proteins significantly associated according to the Ingenuity Pathways Knowledge Base. The markers are highlighted in grey and node properties are indicated by shapes. Interactions between the nodes are solid (confirmed interactions) and dashed (predicted interactions).

Gene pathway analysis indicated that the PD biomarkers are associated with Wnt signaling, muscle inactivity response, pyruvate biosynthesis and vesicle transport (g:Profiler [36] and Ingenuity Systems). In order to understand the potential biological relevance of the biomarkers for PD we used a prediction tool that identified a regulatory network connecting 12 of the 13 markers (Fig. 5A). The network centered on HNF4A and TNF. Both of these transcription factors have been implicated as playing a role in insulin regulation [37], [38]. The APD regulatory network connected 5 markers to the TNF and PTEN signaling pathways, which are involved in neurodegeneration (Figure 5B) [39].

## Discussion

In this study we show that early stage PD is associated with significant gene expression changes in blood that allowed the identification of a biosignature composed of 13 biomarkers that distinguish PD patients from HC and disease controls. We also identify an APD biosignature of 8 biomarkers that distinguish PD from APD patients. To our knowledge these are the first discriminant functions with coherent diagnostic signatures that assign a weight to each of the markers independently for their ability to distinguish PD patients from HC and APD patients.

Gene ontology analysis of the markers identified muscle inactivity, pyruvate biosynthesis and vesicle transport and processing, which have previously been identified as playing a role in PD [40], [41]. This is interesting since oxidative stress and mitochondrial dysfunction play a role in the etiology and/or development of PD and pyruvate protects mitochondria from oxidative stress [41]. In addition, alpha-synuclein, which plays a role in vesicle trafficking, is present in Lewy bodies in neurons of PD patients and is mutated in some hereditary forms of the disease (reviewed in [42]). PD is also characterized by cell death in the substantia nigra. Several of the biomarkers have been implicated as playing a role in cell death including map4k1 [43], pkm2 [44], [45] and prg3 [46]. Further studies are needed to determine if these genes play a role in cell death in PD.

The PD markers validated in this study are different from those identified in earlier studies [26], [27], [28], [29], [31]. These results reflect the diverse nature of the methods used to identify changes in gene expression in blood, including genome-wide microarray screening (A-AFFY-33 and Human genome SpliceArray™ v1.0) and PCR analysis of select transcripts. A thorough search of the Parkinson’s Disease Database (ParkDB) has revealed that the expression of slc14a1 and mpp1 was identified in an earlier study as dysregulated in the blood of PD patients [47]. In the earlier study they were not identified as biomarkers because of the different criteria used for selection [26]. Interestingly, eleven of the PD markers identified in our study were also found to be dysregulated in the brain of PD patients or in models of PD (Table 2). Only wls and znf134 were not previously identified. Together, these results suggest that although PD is thought of as a disease of the central nervous system it may be accompanied by systemic changes.

**Table 2:** Convergence of PD gene expression studies.

Biomarker	Microarray	Tissue	Controls	Expression	References
	A-AFFY-54	Human SNpc	HC	up	[68]
**c5orf4**	A-AFFY-37	Human neuroblastoma cells	DJ-1 RNAi	down	[69]
	Human genome SpliceArray™ v1.0	Human blood	HC, MSA, PSP	up	This study
	A-AFFY-33	Human Brain prefrontal cortex	HC	up	[70]
**macf1**	A-AFFY-34	Human SNpc	HC	up	[71]
	A-AFFY-54	Human SNpc	HC	up	[68]
	Human genome SpliceArray™ v1.0	Human blood	HC, MSA, PSP	up	This study
	A-AFFY-33	Human Brain	HC	down	[70]
**mpp1**	A-AFFY-33	Human blood	HC	down	[26]
	Human genome SpliceArray™ v1.0	Human blood	HC, MSA, PSP	down	This study
	A-AFFY-33	Human SNpc	HC	up	[70]
**znf160**	A-AFFY-34	Human SNpc	HC	up	[71]
	Human genome SpliceArray™ v1.0	Human blood	HC, MSA, PSP	up	This study
**eftud2**	A-AFFY-34	Human SNpc	HC	down	[71]
	Human genome SpliceArray™ v1.0	Human blood	HC, MSA, PSP	up	This study
**prg3**	A-AFFY-33	Human Brain	HC	up	[70]
	Human genome SpliceArray™ v1.0	Human blood	HC, MSA, PSP	up	This study
**pkm2**	A-AFFY-34	Human SNpc	HC	down	[71]
	Human genome SpliceArray™ v1.0	Human blood	HC, MSA, PSP	down	This study
	A-AFFY-34	Human SNpc	HC	down	[71]
**copz1**	A-AFFY-37	Human neuroblastoma cells	DJ-1 RNAi	up	[69]
	Human genome SpliceArray™ v1.0	Human blood	HC, MSA, PSP	up	This study
	A-AFFY-33	Human blood	HC	down	[26]
**slc14a1**	A-AFFY-34	Human SNpc	HC	up	[71]
	Human genome SpliceArray™ v1.0	Human blood	HC, MSA, PSP	down	This study
**map4k1**	A-AFFY-33	Human SNpc	HC	up	[70]
	Human genome SpliceArray™ v1.0	Human blood	HC, MSA, PSP	up	This study

Each marker that had at least a 1.5 fold-change in expression compared to controls (p<0.05) according to ParkDB was included in the analysis [47]. SNpc is substantia nigra pars compacta.

Several of the PD biomarkers are also dysregulated in Alzheimer’s disease including c5orf4, slc14a1, macf1, znf160 and mpp1 [48], [49]. Macf1 is highly expressed in neuronal tissues where it is a positive regulator of Wnt signaling, which is important for axon guidance and synapse formation [50]. In addition, wls regulates the secretion of Wnt proteins, which play important roles in neuronal development [51] and synaptic remodeling [52]. Prg3 is highly expressed in the brain where it promotes neurite growth [53]. Copz1 and map4k1 are also dysregulated in amyotrophic lateral sclerosis patients [54], [55].

Network analysis revealed a regulatory network connecting all of the biomarkers whose functions are known to the transcription factors HNF4A and TNF, which are involved in insulin regulation [37], [38]. In addition, mpp1 and pkm2 are associated with type 1 and 2 diabetes, respectively [56], [57]. This is interesting in light of the fact that patients with diabetes mellitus may have an increased risk of developing PD [58] and more than 60% of the PD patients have impaired insulin signaling and are glucose intolerant [59]. In contrast, others have found an inverse relationship between PD and diabetes preceding PD onset [60]. In addition, mitochondrial dysfunction, endoplasmic reticulum stress, abrogation of the ubiquitin-proteasome and autophagy-lysosome systems and inflammation are involved in the etiology and/or progression of both diseases. One possible explanation for the similarities is that alterations in metabolism in response to environmental factors such as poor dietary practices, heavy metals and pesticides, may lead to insulin resistance, which later develops into diabetes and/or neurodegeneration. In this regard, glucose deprivation induces the aggregation of α-synuclein in dopaminergic cells and leads to cell death [59]. In addition, defects in glucose utilization and sensing occur early in PD pathogenesis [3]. Further studies are needed to determine if there is a correlation between insulin resistance and PD.

The identification of PD splice variant biomarkers suggests that the expression of some splicing regulatory factors is disrupted in early stages of the disease. The expression of SRRM2 and several other splicing factors were previously shown to be disrupted in whole blood and brains of PD patients [31], [61]. The biomarker eftud2, which encodes the splicing factor U5-116 kD, may now be added to this list. Haploinsufficiency of eftud2 causes mandibulofacial dysostosis with microcephaly, a rare syndrome characterized by mental retardation [62]. The identification of eftud2 and the other splice variant markers in this study provides a foundation for future studies directed at understanding mechanistic changes in gene expression that occur at the onset of PD. In this regard, disruption of proteosome function and oxidative stress are associated with PD. A recent study showed that mild proteasome inhibition affects alternative splicing [63]. In addition, oxidative stress disrupts the regulation of alternative splicing of CD44 and the splicing factor transformer ß [64]. Blood expression profiling of splice variants has identified a biosignature for Alzheimer’s disease in which there is no overlap in the markers identified in this study [65]. The identification of highly sensitive and splice variant-specific expression profiles in AD, PD and APD suggests that this approach may be useful for studying other neurodegenerative diseases for which biomarkers are needed.

There are several limitations that should be kept in mind when interpreting these results. Although there were 124 participants in this study, additional patient populations need to be studied to evaluate the generality of these findings. In addition, although strict standards were followed in making the case diagnoses, collecting, processing and analyzing the samples, the results may be vulnerable to bias from unanticipated confounds or diagnostic error. In addition, technical bias and overfitting may occur and, therefore, the biomarkers must be further tested in a larger patient population. In order to determine whether the biomarkers are useful for detecting pre-symptomatic PD, samples from a longitudinal study will need to be tested [66]. Further study of the biomarkers identified here is expected to facilitate the early identification and treatment of this devastating illness.

## Materials and Methods

### Subjects

The Institutional Review Boards of University of Rochester School of Medicine and Rosalind Franklin University of Medicine and Science approved the study protocol. Written informed consent was received from all participants. 124 individuals including 51 PD patients (Hoehn and Yahr scale 1–2) and 39 healthy HC, 17 MSA and 17 PSP age-matched controls were enrolled in the Prognostic Biomarker Study (#NCT00653783). The parent cohort for the PD patients from the PRECEPT, PostCEPT and LAB-PD studies has previously been described [32]. Participant data (age, gender, PD severity score, medications and diabetes status) is presented in Table 1. Criteria used for inclusion/exclusion of participants and for clinical diagnosis used by neurologist trained in movement disorders are presented in Table S1). Clinical diagnosis of PD was based on the United Kingdom Parkinson’s Disease Society Brain Bank criteria requiring the presence of two cardinal features and at least three supportive features [33]. The HC had no history of neurological disease and a Mini-Mental State Examination (MMSE) test score that was ≥27. A diagnosis of probable MSA was based on Consensus Criteria [34] and probable PSP based on NINDS-PSP Criteria [35].

### RNA Extraction and Quality Control

Whole blood (20 ml) was collected in the morning between 8 am and 12 pm during the baseline visit using the PAXgene Blood RNA system (Qiagen,Valencia,CA). The tube was inverted 8–10 times and incubated at room temperature of 24 h. The blood samples were frozen at −20°C until processed for total RNA isolation. Samples from PD patients were processed in parallel with those of controls. RNA was extracted using the PAXgene blood RNA kit according to the manufacturer’s protocol followed by DNase I digestion. RNA quality was determined using the RNA 6000 NanoChip kit and an Agilent 2100 Bioanalyzer (Agilent Technologies, Santa Clara, CA). Samples with RNA integrity values >7.0 and absorbance 260/280 between 1.7 and 2.4 were used.

### Microarray Procedures

Amplified and labeled cDNA was prepared using the NuGEN WT-Ovation™ Pico RNA Amplification System and the FL-Ovation™ cDNA Biotin Module V2 (NuGEN, CA). ExonHit Therapeutics, Inc (Gaithersburg, MD) prepared cDNA from total RNA and the DNA/RNA heteroduplex was amplified by SPIA™ (NuGEN™, San Carlos, CA) [67]. The RNA 6000 Nano kit was used to evaluate the quality of the cDNA. Standard methods were used to hybridize the samples to the Human Genome Wide SpliceArray™, v1.0 (ExonHit Therapeutics, Inc) following recommendations of the manufacturer (Affymetrix, Santa Clara, CA). The arrays were stained and washed using the FS450-001 fluidics protocol prior to scanning with the GeneChip® Scanner 3000 7G (Affymetrix).

### Microarray Data Analysis

Data analysis was performed with Partek GS 6.5 software (St. Louis, MO). The microarray results comply with MIAME guidelines. An analysis of variance (ANOVA) was performed to compare each group of study participants. 10,563 probes were identified that produced a signal above background that was at least 2-fold changed (p-value <0.05). The following three analytical steps were used to identify markers that distinguish PD from controls (Fig. 1A).

#### Step 1

Identification of splice variants that are differentially expressed in PD patients compared to each control group. A Venn diagram tool was used to identify splice events that were differentially expressed in the PD patients compared to each control group (PD vs HC, PD vs MSA and PD vs PSP). The union of the splice events identified by this comparison included 61 splice variants (Fig. 2A).

#### Step 2

Identification of splice variants that are differentially expressed in PD patients compared to non-PD controls. Another ANOVA was performed to compare PD to the controls, without distinguishing between the type of control. Seventeen splice variants were identified, of which 12 were new candidates (Fig. 2A).

#### Step 3

Identification of splice variants with optimized accuracy of prediction. We applied a two-level nested LOOCV. An “outer” 39-fold cross-validation was performed to estimate prediction error of the classifier while a nested, “inner”, 38-fold cross-validation was performed to select the best performing classifier. We assessed K-Nearest Neighbor classification models with the number of neighbors 3, 5, 7 and 9 and number of variables from 1 to 12 using an ANOVA. The best performing classification model contained 12 splice events (Fig. 2A).

### Real-Time PCR

Candidate risk markers were prioritized based on the role they may play in PD etiology or progression based on information from pathway and disease analysis using Ingenuity (Ingenuity Systems, Inc. Redwood City, CA) software. The sequence of the splice variants was retrieved from the UCSC genome browser (http://genome.ucsc.edu/). Splice variant-specific primers were designed using Primer Express software (Applied Biosystems,Foster City,CA) such that one of the primers spanned the splice junction. The High Capacity RNA transcription kit (Applied Biosystems, Foster City,CA) was used to reverse transcribe 1µg of total RNA according to the manufacturer’s protocol. The sequence of the primers and the number of cycles used to amplify the products is presented in Table S2. The region of the transcript amplified for each risk marker is shown in Figure S1. The DNA engine Opticon 2 Analyzer (Bio-Rad Life Sciences, Hercules, CA) was used for the qPCR reactions. Each 25 µl reaction contained Power SYBR and primers at a concentration of 0.05 µM. The amplification conditions used are as follows: denature at 95°C for 15 sec, annealing at 56°C for 1 min, extension at 72°C for 30 sec for 40–50 cycles of amplification and a 7 min extension at 68°C. Following the PCR reaction a melting curve analysis was run to confirm that a single product was amplified. PCR products were also run on 2% agarose gels and sequenced to verify specificity. Gapdh was used as an internal control. Samples were loaded in triplicate. No cDNA template and PD, HC, PSP and MSA controls were run in every experiment. Amplification efficiencies were higher than 90% for each primer set. Expression data was analyzed using the ΔΔCt method.

### Statistical Analysis

To assess the correlation between expression of splice variants with binary diagnostic categories (PD vs. controls) we calculated the Pearson correlation coefficient for all splice events represented on the microarrays. To assess the prediction accuracy of the set of 13 risk markers we performed LOOCV analysis on the microarray data for these splice events. A Student t test and one-way ANOVA and tukey-kramer post-hoc analysis was used to compare groups in the analysis of the qPCR data using GraphPad Prism (GraphPad Software, La Jolla, CA). Discriminant analysis was performed with Partek GS 6.5 (St. Louis, MO) and JMP 9.0 (Cary, NC) software.

## Supporting Information

Figure S1 **Regions of the risk markers amplified by PCR.** Boxes represent exons. Green boxes represent the variant region of the mRNA. Forward arrows represent forward primers and reverse arrows represent reverse primers. Broken arrows indicate that the primer was designed to span the splice junction.
(TIF)

Figure S2 **Relative mRNA quantification graphs of the risk markers.** (A) Student’s t test and tukey-kramer post hoc analysis was used to compare PD patients with controls (HC, PSP and MSA). Fold change values relative to a calibrator are displayed with error bars indicating SEM. (B) Student’s t test and tukey-kramer post-hoc analysis was used to compare PD with APD patients. Fold change values relative to a calibrator are displayed with error bars indicating SEM. Gapdh mRNA was used as a control. *p<0.01, **p<0.005, ***p<0.001 and ****p<0.0001. PD is Parkinson’s disease and C is control.
(TIF)

Figure S3 **Frequency distribution of the Pearson correlation coefficient for binary diagnostic categories (PD vs. controls).** Locations of Pearson correlation coefficient (r) values for PD risk markers are shown with arrowheads. These values are either below 10% percentile (−0.2111) or above 90% percentile (0.2045).
(TIF)

Figure S4 **Representative relative mRNA quantification graphs of risk markers from a training set of samples.** Splice variants that were expressed differentially in HC compared to disease participants. A student t test was used to compare groups. *p<0.01, **p<0.005 and ****p<0.0001. Fold change values relative to a calibrator are displayed with error bars indicating SEM. Gapdh mRNA was used as a control. HC is healthy control, PD is Parkinson’s disease, MSA is multiple system atrophy, and PSP is progressive supranuclear palsy.
(TIF)

Figure S5 **Principle components and heat map analysis of the APD biomarkers.** (A) Heat map of the data from the qPCR assay of the biomarkers analyzed using the ΔΔCt method. Each column in the heat map corresponds to a study participant. Each row represents the relative level of abundance of a single splice variant. Each splice variant is denoted by the name of the mRNA. Color scales representing splice variant expression with red representing high abundance relative to the mean abundance; blue representing low abundance relative to the mean abundance; and gray representing no significant change in abundance level between the sample and control. PD patients are indicated by green and APD patients are indicated by yellow. (B) Principle components analysis of 52 samples (training set).
(TIF)

Table S1 **Criteria used for inclusion/exclusion of study participants and for clinical diagnosis.**
(DOC)

Table S2 **Biomarker information.** Identifying information for the mRNA sequences that result from an alternative splicing event that are used to diagnose the presence of Parkinson’s disease in a human patient and the specific forward and reverse primers and cycle number used for amplification. ^1^Numbers represent exons; > < represents between exons; < > represents skipped exon; ^2^Event and identifier numbers are located at http://www.ncbi.nlm.nih.gov/and http://genome.ucsc.edu/cgi-bin/hgGateway.
(DOC)

Table S3 **Raw and standardized canonical discriminant function coefficients for the PD biomarkers.** Discriminant analysis was performed with Statistica 8.0 and JMP 9.0 software.
(DOC)

Table S4 **Discriminant analysis results using the PD biomarkers.** Chi Square Test with successive roots removed. Analysis was performed with Statistica 8.0 software.
(DOC)

Table S5 **Linear discriminant analysis performed on gene expression data from 124 participants.** Sensitivity and specificity values are displayed for the three classification groups.
(DOC)

Table S6 **Standardized and raw canonical coefficients for canonical variables in the discriminant function using the APD biomarkers.**
(DOC)

Table S7 **Discriminant function analysis summary for the APD biomarkers.**
(DOC)

Table S8 **Summary of chi-square distribution and canonical correlation of the APD biomarkers.**
(DOC)

Table S9 **Linear discriminant analysis performed on gene expression data from 85 patients.** Sensitivity and specificity values are displayed for each classification group.
(DOC)
