# Supplementary figures and images for: Biosignatures for Parkinson’s Disease and Atypical Parkinsonian Disorders Patients
Source: PLoS One. 2012 Aug 27;7(8):e43595. doi: 10.1371/journal.pone.0043595 (PMC3428307; doi:10.1371/journal.pone.0043595)

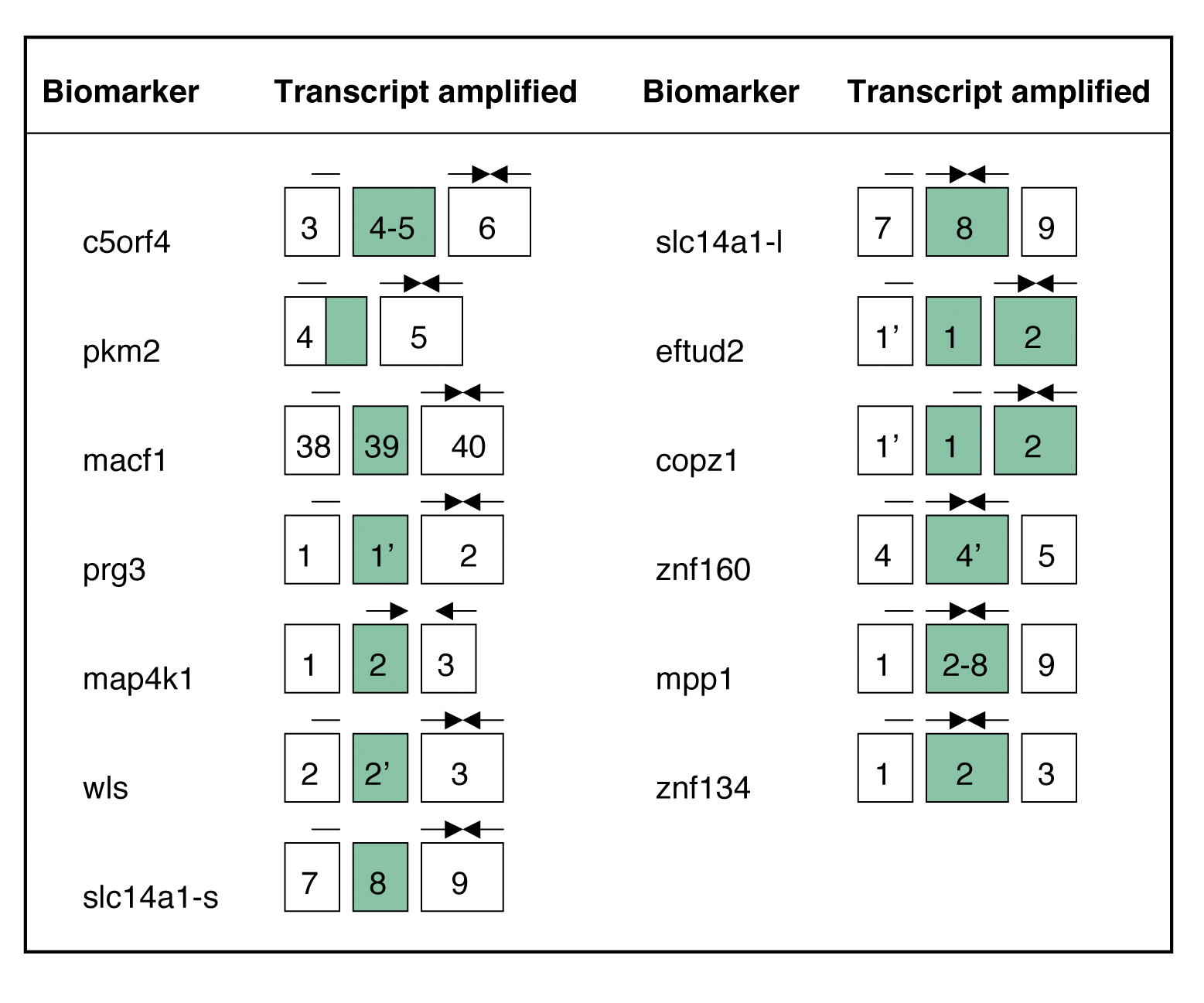

Supplement: Figure S1 — Regions of the risk markers amplified by PCR. Boxes represent exons. Green boxes represent the variant region of the mRNA. Forward arrows represent forward primers and reverse arrows represent reverse primers. Broken arrows indicate that the primer was designed to span the splice junction. (TIF) [file pone.0043595.s001.tif]

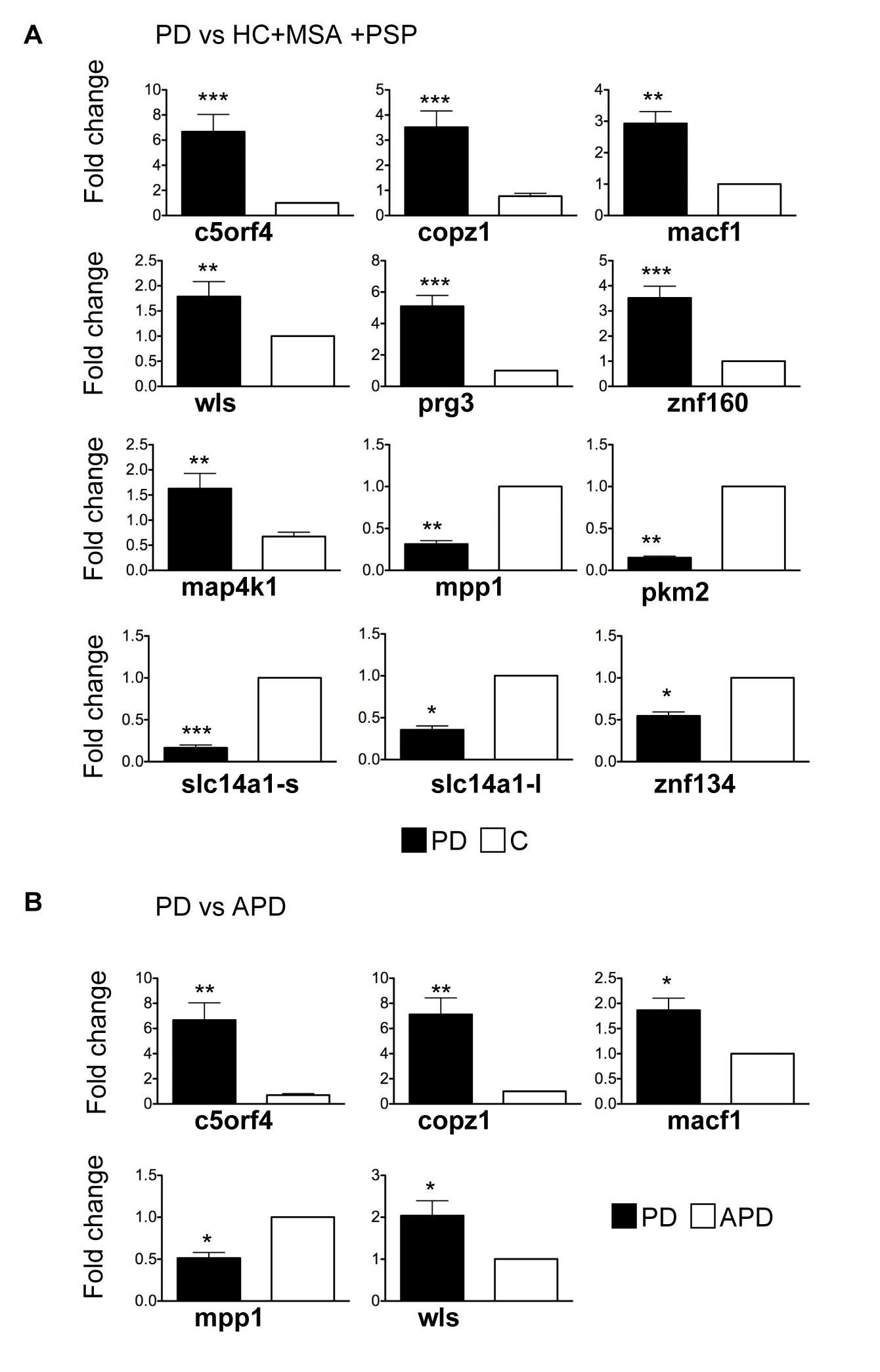

Supplement: Figure S2 — Relative mRNA quantification graphs of the risk markers. (A) Student’s t test and tukey-kramer post hoc analysis was used to compare PD patients with controls (HC, PSP and MSA). Fold change values relative to a calibrator are displayed with error bars indicating SEM. (B) Student’s t test and tukey-kramer post-hoc analysis was used to compare PD with APD patients. Fold change values relative to a calibrator are displayed with error bars indicating SEM. Gapdh mRNA was used as a control. *p<0.01, **p<0.005, ***p<0.001 and ****p<0.0001. PD is Parkinson’s disease and C is control. (TIF) [file pone.0043595.s002.tif]

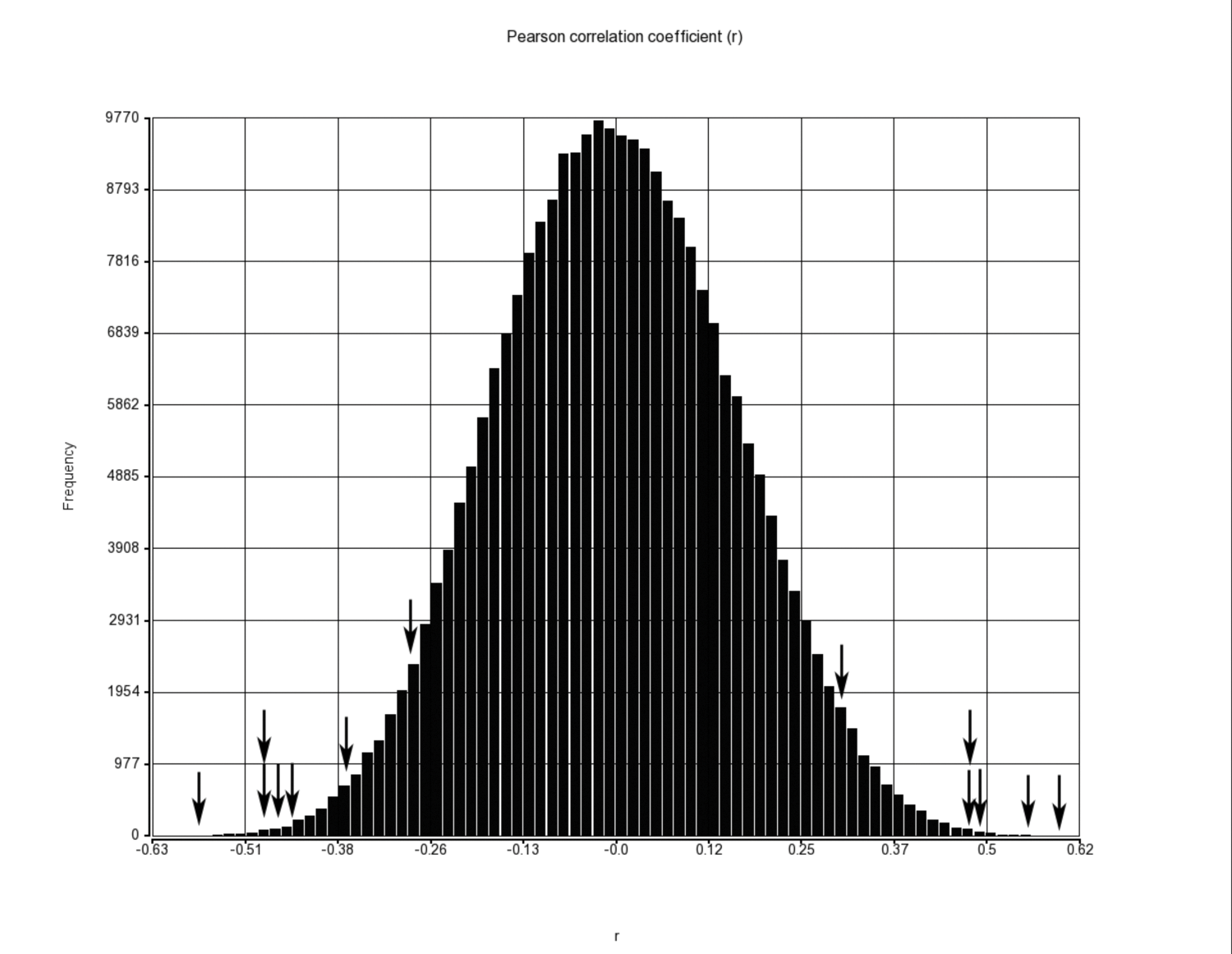

Supplement: Figure S3 — Frequency distribution of the Pearson correlation coefficient for binary diagnostic categories (PD vs. controls). Locations of Pearson correlation coefficient (r) values for PD risk markers are shown with arrowheads. These values are either below 10% percentile (−0.2111) or above 90% percentile (0.2045). (TIF) [file pone.0043595.s003.tif]

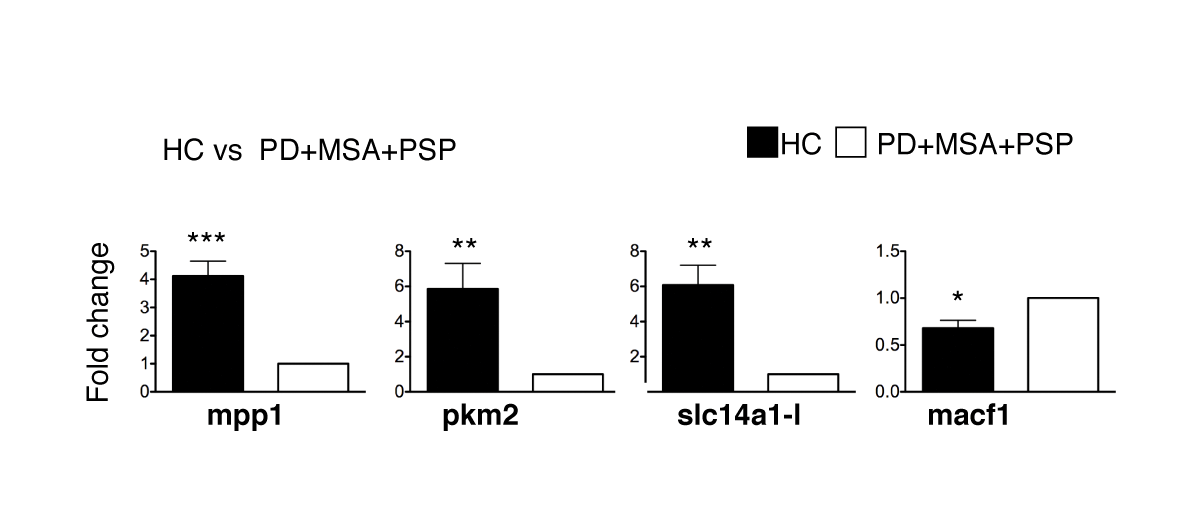

Supplement: Figure S4 — Representative relative mRNA quantification graphs of risk markers from a training set of samples. Splice variants that were expressed differentially in HC compared to disease participants. A student t test was used to compare groups. *p<0.01, **p<0.005 and ****p<0.0001. Fold change values relative to a calibrator are displayed with error bars indicating SEM. Gapdh mRNA was used as a control. HC is healthy control, PD is Parkinson’s disease, MSA is multiple system atrophy, and PSP is progressive supranuclear palsy. (TIF) [file pone.0043595.s004.tif]

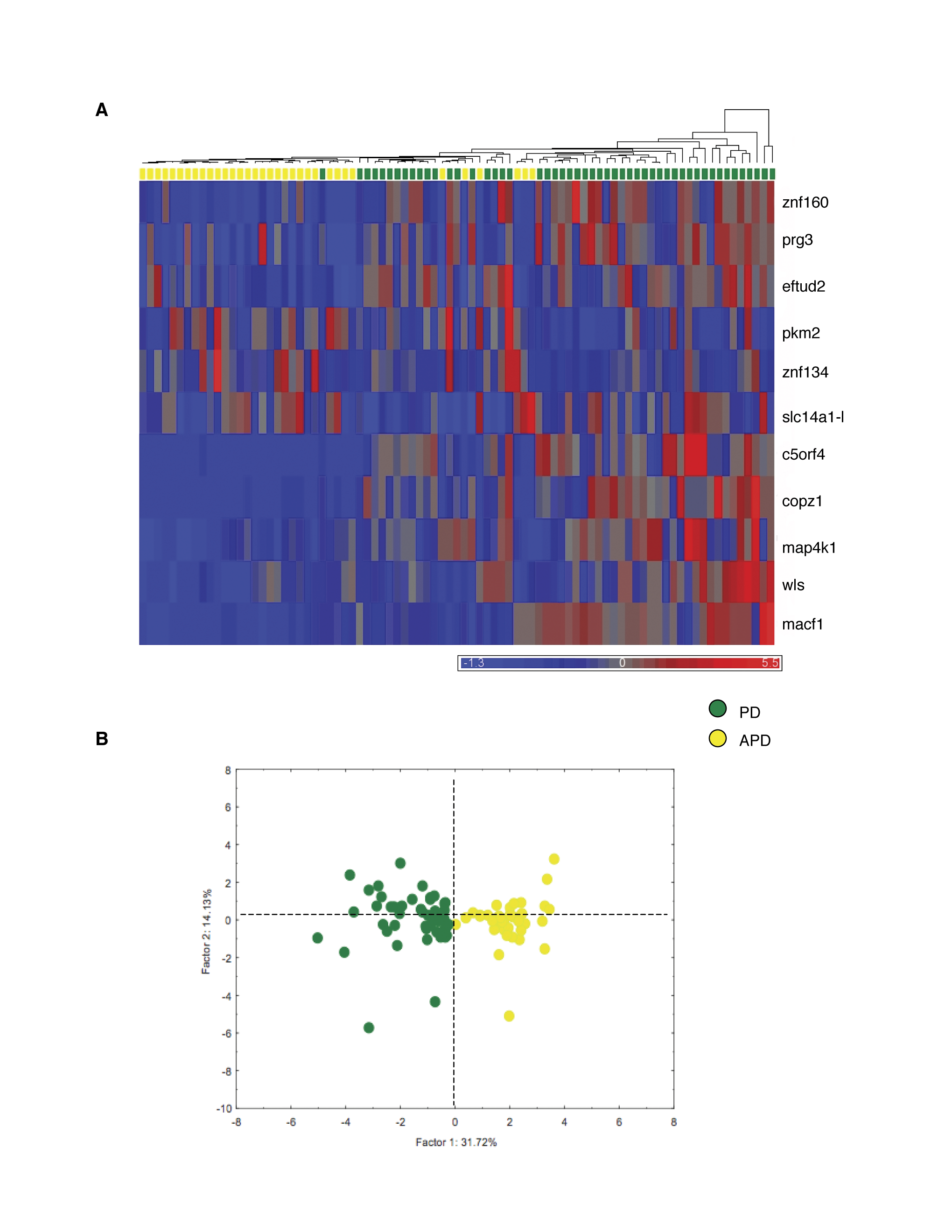

Supplement: Figure S5 — Principle components and heat map analysis of the APD biomarkers. (A) Heat map of the data from the qPCR assay of the biomarkers analyzed using the ΔΔCt method. Each column in the heat map corresponds to a study participant. Each row represents the relative level of abundance of a single splice variant. Each splice variant is denoted by the name of the mRNA. Color scales representing splice variant expression with red representing high abundance relative to the mean abundance; blue representing low abundance relative to the mean abundance; and gray representing no significant change in abundance level between the sample and control. PD patients are indicated by green and APD patients are indicated by yellow. (B) Principle components analysis of 52 samples (training set). (TIF) [file pone.0043595.s005.tif]
