# Supplementary material for: Biosignatures for Parkinson’s Disease and Atypical Parkinsonian Disorders Patients
Source: PLoS One. 2012 Aug 27;7(8):e43595. doi: 10.1371/journal.pone.0043595 (PMC3428307; doi:10.1371/journal.pone.0043595)
Supplement: Table S1 — Criteria used for inclusion/exclusion of study participants and for clinical diagnosis. (DOC) [file pone.0043595.s006.doc]

| **CRITERIA FOR ALL PARTICIPANTS** | |
| --- | --- |
| **Inclusion criteria** | **Exclusion criteria** |
| Willing and able to provide informed consent. | Use of anticoagulants (e.g., warfarin or heparin) or a known bleeding disorder (acquired or inherited);  Known blood disorder (e.g. leukemia) or a history of anemia with documented hematocrit <30;  Known pregnancy. |

| **CRITERIA FOR HEALTHY PARTICIPANTS** |
| --- |
| Spouse or non-blood relative of the PD subject;  No known current diagnosis or history of a neurological disease;  MMSE score >27;  Age >45. |

| **DIAGNOSTIC CRITERIA FOR PD** | | |
| --- | --- | --- |
| **Inclusion criteria** | **Exclusion criteria** | **Supportive criteria** |
| Subject meets UK brain bank criteria for PD including:  Bradykinesia  And at least one of the following:  Muscular rigidity;  4-6 Hz rest tremor;  Postural instability not caused by primary visual, vestibular, cerebellar, or proprioceptive dysfunction. | History of repeated strokes with stepwise progression of parkinsonian features;  History of repeated head injury;  History of definite encephalitis;  Oculogyric crises;  Neuroleptic treatment at onset of symptoms;  More than one affected relative;  Sustained remission;  Strictly unilateral features after 3 years;  Supranuclear gaze palsy;  Cerebellar signs;  Early severe autonomic involvement;  Babinski sign;  Presence of cerebral tumor or communicating hydrocephalus on computed tomography scan;  Negative response to large doses of levodopa (if malabsorption excluded);  MPTP exposure. | (three or more required for diagnosis of definite PD):  Unilateral onset;  Rest tremor present;  Progressive disorder;  Persistent asymmetry affecting side of onset most;  Excellent response (70-100%) to levodopa;  Severe levodopa-induced chorea;  Levodopa response for 5 yr or more;  Clinical course of 10 yr or more. |

| **DIAGNOSTIC CRITERIA FOR MSA** | | |
| --- | --- | --- |
| **Inclusion criteria** | **Exclusion criteria** | **Supportive criteria** |
| A diagnosis of Probable MSA based on Consensus Criteria including:  Autonomic failure involving urinary incontinence (persistent, involuntary partial or total bladder emptying, accompanied by erectile dysfunction in men) or  an orthostatic decrease of blood pressure (within 3 min of standing by 30 mm Hg systolic or 15 mm Hg diastolic) and  Criterion for parkinsonism  Bradykinesia (slowness of voluntary movement with a progressive reduction of speed and amplitude)  plus at least one of the following: rigidity, postural instability (not due to primary visual, vestibular, cerebellar, or proprioceptive dysfunction) or tremor (postural, resting or both);  A cerebellar dysfunction (gait ataxia with cerebellar dysarthria, limb ataxia, or cerebellary oculomotor dysfunction);  Poorly levodopa responsive parkinsonism. | Symptomatic onset before age 30;  Systemic disease or other identifiable cause for feature in inclusion criteria;  Hallucinations unrelated to medication;  Diagnostic and Statistical Manual of Mental Disorders criteria for dementia;  Prominent slowing of vertical saccades or vertical supranuclear gaze palsy;  Evidence of focal cortical dysfunction such as aphasia, alien limb syndrome, and parietal dysfunction. | Babinski sign with hyperreflexia;  Stridor;  Rapidly progressive parkinsonism;  Postural instability within 3 y of motor onset;  Gait ataxia, cerebellar dysarthria, limb ataxia or cerebellar oculomotor dysfunction;  Dysphagia within 5 y of motor onset. |

| **DIAGNOSTIC CRITERIA FOR PSP** | | |
| --- | --- | --- |
| **Inclusion criteria** | **Exclusion criteria** | **Supportive criteria** |
| A diagnosis of Probable PSP based on NINDS-PSP Criteria:  Gradually progressive disorder with age at onset at 40 or later;  Vertical supranuclear palsy and prominent postural instability with falls or tendancy to fall (subjects are not able to stabilize themselves) within first year of disease onset. | Recent history of encephalitis;  Alien limb syndrome;  Cortical sensory deficits;  Focal frontal or temporoparietal atrophy;  Hallucinations or delusions unrelated to dopaminergic therapy;  Cortical dementia of Alzheimer type;  Prominent, early cerebellar symptoms or unexplained dysautonomia; or evidence of other diseases that could explain the clinical features. | Symmetric akinesia or rigidity, proximal more than distal;  Abnormal neck posture, especially retrocollis;  Poor or absent response of parkinsonism to levodopa;  Early dysphagia and dysarthria;  Early onset of cognitive impairment including >2 of: apathy,  Impairment in abstract thought, decreased verbal fluency, utilization or imitation behavior, or frontal release signs. |
