# Supplementary material for: Biosignatures for Parkinson’s Disease and Atypical Parkinsonian Disorders Patients
Source: PLoS One. 2012 Aug 27;7(8):e43595. doi: 10.1371/journal.pone.0043595 (PMC3428307; doi:10.1371/journal.pone.0043595)
Supplement: Table S2 — Biomarker information. Identifying information for the mRNA sequences that result from an alternative splicing event that are used to diagnose the presence of Parkinson’s disease in a human patient and the specific forward and reverse primers and cycle number used for amplification. 1Numbers represent exons; > < represents between exons; < > represents skipped exon; 2Event and identifier numbers are located at http://www.ncbi.nlm.nih.gov/and http://genome.ucsc.edu/cgi-bin/hgGateway. (DOC) [file pone.0043595.s007.doc]

| **Event No.^2^** | **Name** | **Event location^1^** | **Identifier^2^** | **5’ (Forward)** | **3’ (Reverse)** | **Cycles** |
| --- | --- | --- | --- | --- | --- | --- |
| 5315.014.2 | pkm2  Alternative  Splice donor | 4 | NM_182471.1 | CACAGCCAGCCGACTTCCT | TTCAGATCCTGGATGTCCTTCTC | 40 |
| 10826.005.1 | c5orf4  Exon skipped | 4< >5 | BP346979.1 | GACATGGTGGATCCTGTGAAACT | GAAAGATATCATGCACTGGTTGAAA | 50 |
| 23499.058.1 | macf1  Exon skipped | 39< >39 | EH_012090.39 | ATGAGGCGCTCCAGGAAGA | AGGTCACTGCTTCCTCCAGTTC | 50 |
| 54886.003.1 | prg3  Novel exon | 1> <2 | AX050009.1 | CGGGAGCCTGGACAGTTTT | CCTACAGCCATTTCTCACACCAT | 40 |
| 11184.010.1 | map4k1  Exon skipped | 2< >2 | NM_007181.3 | GGACCTGGTGGCACTGAAGA | CGGCAAGTTTTCAATATGAGGAT | 40 |
| 79971.007.1 | wls  Novel exon | 2> <3 | DB035783.1 | CAAGCTAAACAACCAAATCAGAGAAA | ATCACGGTAAGCCAGGGAAA | 40 |
| 6563.016.1 | slc14a1-s  Exon skipped | 8< >8 | NM_015865.1 | CACTCATGTGCCTGCATGCT | AACAGGGCCGCTGCTATG | 50 |
| 6563.016.1 | slc14a1-l  Exon included | 8 | EH_015865.1 | GCATAGCAGCGGGACTCAGT | ACCCCAGAGTCCAAAGTAGATGTC | 50 |
| 9343.019.2 | eftud2  Exon skipped | 1< >1 | NM_004247.2 | AGCAGGCGAGAGATGGATGA | CGGCTGTTGGGTAGTACTTCTTG | 50 |
| 22818.003.2 | copz1  Exon skipped | 1< >1 | NM_016057.1 | GATTTTGTGGTGGGAAAGAGT | TGACAGCTCCCCTAGATCTTTG | 40 |
| 90338.007.1 | znf160  Novel Exon | 4> <5 | DB292168.1 | GGGTCGCTACGGACTTAAAATC | CGGATCCAGGAACGTTTCTG | 40 |
| 4354.006.1 | mpp1  Exon skipped | 2< >8 | NM_002436.2 | GAGGCTGTATCGCATCCATTG | GCAGGAGACCCGTTGGTGTA | 40 |
| 7693.001.1 | znf134  Exon skipped | 2< >2 | NM_003435.2 | GGCCCGGCGCAGAT | GCAGCCAGAGTTCTCTCTCTTGT | 40 |
| Control | gapdh |  | NM_002046.3 | CAACGGATTTGGTCGTATTGG | TGATGGCAACAATATCCACTTTACC | 40 |
