# Supplementary material for: Biosignatures for Parkinson’s Disease and Atypical Parkinsonian Disorders Patients
Source: PLoS One. 2012 Aug 27;7(8):e43595. doi: 10.1371/journal.pone.0043595 (PMC3428307; doi:10.1371/journal.pone.0043595)
Supplement: Table S3 — Raw and standardized canonical discriminant function coefficients for the PD biomarkers. Discriminant analysis was performed with Statistica 8.0 and JMP 9.0 software. (DOC) [file pone.0043595.s008.doc]

| **Biomarkers** | **Raw coefficients** | **Standardized coefficients** |
| --- | --- | --- |
| c5orf4 | -1.39E+02 | -0.694 |
| macf1 | -1.40E+01 | -0.416 |
| copz1 | -4.40E+01 | -0.386 |
| znf160 | -6.69E+06 | -0.335 |
| mpp1 | 2.00E+00 | 0.508 |
| pkm2 | -9.00E+00 | -0.179 |
| znf134 | 9.20E+01 | 0.286 |
| slc14a1-s | 1.74E+04 | 0.279 |
| wls | 8.00E+00 | 0.228 |
| prg3 | -6.52E+04 | -0.085 |
| map4k1 | -1.00E+00 | -0.034 |
| slc14a1-l | 1.00E+00 | 0.144 |
| eftud2 | -2.35E+04 | -0.280 |
| Eigenvalue | 3 | 2.78 |
