# Supplementary material for: Biosignatures for Parkinson’s Disease and Atypical Parkinsonian Disorders Patients
Source: PLoS One. 2012 Aug 27;7(8):e43595. doi: 10.1371/journal.pone.0043595 (PMC3428307; doi:10.1371/journal.pone.0043595)
Supplement: Table S4 — Discriminant analysis results using the PD biomarkers. Chi Square Test with successive roots removed. Analysis was performed with Statistica 8.0 software. (DOC) [file pone.0043595.s009.doc]

| **Eigenvalue** | **Canonical R** | **Wilks' Lambda** | **Chi-Sqr.** | **df** | **p-level** |
| --- | --- | --- | --- | --- | --- |
| 2.788944 | 0.857948 | 0.263926 | 153.8561 | 13.00000 | 0.001 |
