# Supplementary material for: Biosignatures for Parkinson’s Disease and Atypical Parkinsonian Disorders Patients
Source: PLoS One. 2012 Aug 27;7(8):e43595. doi: 10.1371/journal.pone.0043595 (PMC3428307; doi:10.1371/journal.pone.0043595)
Supplement: Table S6 — Standardized and raw canonical coefficients for canonical variables in the discriminant function using the APD biomarkers. (DOC) [file pone.0043595.s011.doc]

| **APD Biomarker** | **Standardized canonical discriminant function coefficients** | **Raw coefficients for canonical variables** |
| --- | --- | --- |
| copz1 | -0.545 | -51.76 |
| c5orf4 | -0.991 | -169.11 |
| mpp1 | 0.573 | 3.08 |
| macf1 | -0.784 | -22.09 |
| wls | 0.458 | 14.85 |
| slc14a1-l | 0.544 | 11.39 |
| znf134 | 0.190 | 72.16 |
| map4k1 | -0.168 | -5.50 |
| Constant | 1.989 | 0.13 |
| Eigenvalue | -0.545 | 1.99 |
