# Supplementary material for: Biosignatures for Parkinson’s Disease and Atypical Parkinsonian Disorders Patients
Source: PLoS One. 2012 Aug 27;7(8):e43595. doi: 10.1371/journal.pone.0043595 (PMC3428307; doi:10.1371/journal.pone.0043595)
Supplement: Table S7 — Discriminant function analysis summary for the APD biomarkers. (DOC) [file pone.0043595.s012.doc]

| **APD Biomarker** | **Wilks Lambda** | **Partial Lambda** | **F-remove (1,45)** | **p-level** | **tolerance** | **1-tolerance (R-sqr)** |
| --- | --- | --- | --- | --- | --- | --- |
| copz1 | 0.395246 | 0.846249 | 13.80810 | 0.000385 | 0.777684 | 0.222316 |
| c5orf4 | 0.523565 | 0.638843 | 42.96508 | 0.000000 | 0.552152 | 0.447848 |
| mpp1 | 0.379404 | 0.881582 | 10.20862 | 0.002036 | 0.541027 | 0.458973 |
| macf1 | 0.435399 | 0.768207 | 22.93174 | 0.000008 | 0.565895 | 0.434105 |
| wls | 0.365146 | 0.916005 | 6.96897 | 0.010059 | 0.601461 | 0.398539 |
| slc14a1-l | 0.365096 | 0.916131 | 6.95756 | 0.010118 | 0.424904 | 0.575096 |
| znf134 | 0.342040 | 0.977887 | 1.71857 | 0.193824 | 0.914975 | 0.085025 |
| map4k1 | 0.339063 | 0.986473 | 1.04218 | 0.310554 | 0.713404 | 0.286596 |
