# Supplementary material for: Biosignatures for Parkinson’s Disease and Atypical Parkinsonian Disorders Patients
Source: PLoS One. 2012 Aug 27;7(8):e43595. doi: 10.1371/journal.pone.0043595 (PMC3428307; doi:10.1371/journal.pone.0043595)
Supplement: Table S8 — Summary of chi-square distribution and canonical correlation of the APD biomarkers. (DOC) [file pone.0043595.s013.doc]

| **Eigenvalue** | **Canonical R** | **Wilk’s Lambda** | **Chi-sqr** | **df** | **p-level** |
| --- | --- | --- | --- | --- | --- |
| 1.99 | 0.82 | 0.33 | 86.5 | 8 | 0.0001 |
