# Supplementary material for: Biosignatures for Parkinson’s Disease and Atypical Parkinsonian Disorders Patients
Source: PLoS One. 2012 Aug 27;7(8):e43595. doi: 10.1371/journal.pone.0043595 (PMC3428307; doi:10.1371/journal.pone.0043595)
Supplement: Table S9 — Linear discriminant analysis performed on gene expression data from 85 patients. Sensitivity and specificity values are displayed for each classification group. (DOC) [file pone.0043595.s014.doc]

| **Diagnosis** | **Total (N)** | **Correctly Classified** | **Sensitivity/Specificity** |
| --- | --- | --- | --- |
| PD | 51 | 96 | 96/90 |
| APD | 34 | 94 | 94/96 |
